# Supplementary material for: Viperin controls chikungunya virus–specific pathogenic T cell IFNγ Th1 stimulation in mice
Source: Life Sci Alliance. 2019 Jan 21;2(1):e201900298. doi: 10.26508/lsa.201900298 (PMC6342136; doi:10.26508/lsa.201900298)
Supplement: Supplementary file 1 [file LSA-2019-00298_TableS1.pdf]

Table S1: Reagent and antibodies used for immune phenotyping by flow cytometry.

**Marker**	**Color**	**Dilution**	**Manufacturer (Reference)**
Live Dead stain	Fixable Aqua Dead	1:400	Thermofisher (L34966)
CD45	BUV395	1:400	BD (564279)
CD4	PacBlue	1:400	Biolegend (100531)
CD8	CF594	1:400	BD (562283)
CD3	PE-Cy7	1:200	Biolegend (100220)
Ly6C	APC-Cy7	1:400	Biolegend (128026)
MHC-II	AF700	1:400	Biolegend (107622)
LFA-1	PerCP-Cy5.5	1:400	Biolegend (141008)
CD11b	BV650	1:400	Biolegend (101239)
CD11c	BV605	1:400	Biolegend (117333)
Ly6G	CF594	1:400	BD (562700)
B220	eFluor450	1:400	ebioscience (48-0452-82)
MerTK	PE	1:400	ebioscience (12-5751-82)
CD64	APC	1:200	Biolegend (139306)
NK1.1	Biotin	1:200	ebioscience (13-5941-85)
Streptavidin	BUV737	1:200	BD (564293)
CD45.1	PE	1:400	BioLegend (110707)
CD45.2	APC	1:400	Ebioscience (17-0454082)
