# Supplementary material for: Viperin controls chikungunya virus–specific pathogenic T cell IFNγ Th1 stimulation in mice
Source: Life Sci Alliance. 2019 Jan 21;2(1):e201900298. doi: 10.26508/lsa.201900298 (PMC6342136; doi:10.26508/lsa.201900298)
Supplement: Supplementary file 2 [file LSA-2019-00298_TableS2.pdf]

Table S1: Reagent and antibodies used for T-cell phenotyping by flow cytometry. Markers in italics are intracellular staining.

**Marker**	**Color**	**Dilution**	**Manufacturer (Reference)**
Live Dead stain	Fixable Aqua Dead	1:400	Thermofisher (L34966)
NK1.1	Biotin	1:400	ebioscience (13-5941-85)
CD19	Biotin	1:400	Biolegend (115504)
Ter119	Biotin	1:400	Biolegend (116204)
Streptavidin	BUV737	1:200	BD (564293)
CD45	BUV395	1:400	BD (564279)
CD4	PacBlue	1:200	Biolegend (100531)
CD3	AF488	1:200	Biolegend (100220)
CD44	APC-Cy7	1:400	Biolegend (103208)
IFNγ	PerCP-Cy5.5	1:200	ebioscience (45-7311-82)
IL4	PE	1:200	Biolegend (504104)
IL-17A	APC	1:200	ebioscience (17-7177-81)
TFNα	BV650	1:200	BD (563943)
IL-10	BV605	1:200	Biolegend (505031)
CCR6	BV605	1:400	Biolegend (129819)
CCR4	PE-Cy7	1:400	Biolegend (131214)
CXCR3	PerCP-Cy5.5	1:200	ebioscience (45-1831-82)
Tbet	PE	1:100	ebioscience (12-5825-82)
RoRγ*t*	APC	1:100	ebioscience (17-6988-82)
GATA3	BV711	1:100	BioLegend (110707)
